# Mechanically Soft Phase-Separated Gelatin/Hyaluronic Acid Hydrogels Support Long-Term Expansion of Human Mesenchymal Stem Cells While Preserving Multipotency

**DOI:** 10.3390/ijms27072932

**Published:** 2026-03-24

**Authors:** Atsushi Yamashita, Nunnarpas Yongvongsoontorn, Joo Eun Chung, Motoichi Kurisawa

**Affiliations:** 1 Institute of Bioengineering and Bioimaging, 31 Biopolis Way, The Nanos, Singapore 138669, Singapore; 2 Graduate School of Advanced Science and Technology, Japan Advanced Institute of Science and Technology, 1-1 Asahidai, Nomi 923-1292, Ishikawa, Japan

**Keywords:** soft substrates, phase-separated hydrogels, a-smooth muscle actin, long-term expansion, mesenchymal stem cells

## Abstract

Large-scale expansion of human mesenchymal stem cells (hMSCs) remains a major challenge due to the intrinsic trade-off between cell proliferation and the maintenance of multipotency in conventional culture systems. Stiff substrates, such as tissue culture polystyrene or rigid hydrogels, promote rapid proliferation but induce progressive loss of stemness, whereas very soft matrices preserve multipotency at the expense of cell growth. To overcome this limitation, we developed mechanically soft, phase-separated gelatin–phenol/hyaluronic acid–phenol (Gtn-Ph/HA-Ph) hydrogels with precisely controlled microstructures via enzyme-mediated crosslinking. These hydrogels consist of HA-rich, dot-like domains embedded within a continuous Gtn-rich network, allowing for independent tuning of stiffness and domain architecture. On single-component Gtn-Ph hydrogels, hMSC proliferation increased with substrate stiffness, whereas soft hydrogels with a storage modulus (*G*′) of approximately 0.6 kPa markedly suppressed proliferation while preserving stemness marker expression, confirming the stiffness-dependent trade-off. In contrast, phase-separated Gtn-Ph/HA-Ph hydrogels supported robust hMSC proliferation even under soft mechanical conditions while maintaining high expression of stemness-associated markers. During long-term culture, hMSCs achieved a 68- to 195-fold increase in cumulative cell yield on soft Gtn-Ph/HA-Ph hydrogels (*G*′ = 0.5 kPa) compared with tissue culture polystyrene. Expression of α-smooth muscle actin (α-SMA) mRNA, encoded by the *ACTA2* gene and associated with cellular senescence and fibrotic activation, was completely suppressed, while hMSCs retained robust adipogenic, osteogenic, and chondrogenic differentiation capacities. These results demonstrate that phase-separated Gtn-Ph/HA-Ph hydrogels effectively resolve the proliferation–multipotency dilemma in hMSC expansion and provide a promising platform for scalable manufacturing of therapeutic stem cells.

## 1. Introduction

Human mesenchymal stem cells (hMSCs) are widely recognized as a promising cell source for regenerative medicine owing to their self-renewal capacity, multilineage differentiation potential, and immunomodulatory properties. Over the past few decades, numerous hMSC-based therapies have been applied for clinical applications, including the treatment of degenerative arthritis, Crohn’s disease, myocardial infarction, and graft-versus-host disease (GVHD) [1,2,3,4,5]. In addition, a broad range of indications, such as chronic wounds, neurological injuries, pulmonary failure, liver cirrhosis, and diabetes mellitus, are currently under investigation in clinical trials [3]. With the rapid expansion of these translational efforts, the establishment of efficient and scalable hMSC expansion systems has become an essential prerequisite for both clinical implementation and industrial manufacturing [6,7,8,9,10,11]. Despite their broad therapeutic potential, hMSCs progressively lose proliferative capacity and multipotency during prolonged in vitro expansion, particularly with increasing passage number. To mitigate this limitation, various culture media formulations have been developed, including fetal bovine serum (FBS)-containing [12,13,14], xeno-free [15,16,17], and animal component-free (ACF) media [18,19,20,21]. Among these, ACF media are particularly attractive for clinical applications because they eliminate both animal- and human-derived components. Nevertheless, conventional tissue culture polystyrene (TCPS) dishes and flasks remain the standard substrates for hMSC expansion. Although such stiff substrates (~2 GPa) support rapid proliferation of early-passage hMSCs, they also induce mechanical activation of the cells, leading to increased expression of α-smooth muscle actin (α-SMA), a hallmark of myofibroblastic transition and hMSC senescence, along with reduced clonogenicity and loss of multipotency [22,23,24]. Therefore, the development of culture substrates capable of suppressing α-SMA expression during long-term expansion remains a critical unmet need.

In contrast to stiff substrates, soft substrates with a storage modulus (*G*′) of 6–15 kPa have been shown to inhibit α-SMA expression and better preserve hMSC multipotency [25]. Similar mechanical regulation has been reported in other cell types, including embryonic stem cells and cancer cells, where very soft hydrogels (*G*′ = 0.3–1.8 kPa) promote pluripotency-associated or cancer stem cell-like properties, respectively [26,27,28]. However, excessively soft substrates generally fail to support efficient cell proliferation, as reduced cell contractility and impaired integrin-mediated signaling suppress cell growth and cell cycle progression [29,30,31,32]. Consequently, a fundamental dilemma exists between promoting hMSC proliferation and preserving multipotency, representing a major bottleneck in the scalable manufacturing of clinical-grade hMSCs. Beyond bulk substrate stiffness, hMSC behavior is strongly influenced by the chemical composition and microarchitecture of the culture surface [32]. Extracellular matrix (ECM) proteins such as collagen, gelatin (Gtn), fibronectin, and laminin enhance cell adhesion through integrin-mediated interactions and regulate cell survival and proliferation [33,34,35,36,37]. Furthermore, the spatial organization of adhesive regions, including their size and spacing, plays a critical role in controlling cell spreading, apoptosis, differentiation, and proliferation [38,39]. Notably, micro-patterned adhesive domains with reduced feature sizes have been reported to enhance cell proliferation while suppressing apoptosis, suggesting that microstructural design can be leveraged to decouple cell proliferation from bulk substrate stiffness. Although photolithographic techniques have been widely used to fabricate precisely defined ECM micropatterns [40,41], these approaches typically require costly instrumentation, photomasks, and complex processing steps, limiting their scalability and accessibility. In addition to organizing adhesive ECM proteins, polysaccharides such as hyaluronic acid (HA), a major glycosaminoglycan component of native ECM, play essential roles in regulating stem cell behavior. HA interacts with CD44 receptors and contributes to the maintenance of stemness and self-renewal in hMSCs [42,43,44,45,46]. HA coatings on TCPS [43] and HA-based three-dimensional hydrogels [47] have both been shown to preserve hMSC multipotency. These findings suggest that combining integrin-adhesive protein micropatterns with HA-rich domains on soft substrates could provide a biomimetic microenvironment that may support both proliferation and stemness. However, although Gtn and HA-based composite hydrogels have been explored for hMSC culture and differentiation [48,49], the potential of phase-separated Gtn/HA hydrogels to support large-scale hMSC expansion while preserving long-term multipotency remains largely unexplored.

In this study, we present an effective strategy for resolving the long-standing proliferation–multipotency dilemma in hMSC culture by fabricating micrometer-scale phase-separated hydrogel surfaces. This approach exploits an aqueous two-phase system (ATPS), in which two incompatible water-soluble polymers spontaneously separate into distinct phases at appropriate concentrations [50,51,52,53]. By harnessing the time-dependent phase separation behavior of ATPS during enzymatic crosslinking, we fabricated Gtn-phenol (Gtn-Ph)/HA-phenol (HA-Ph) hydrogels with micrometer-scale HA-rich domains embedded within a continuous Gtn-rich network. The domain size and spatial distribution were precisely controlled by adjusting the concentration of horseradish peroxidase (HRP), which governs the crosslinking kinetics. Importantly, this phase-separated architecture is fundamentally distinct from previously reported Gtn and HA-based composite hydrogels. By introducing microstructural heterogeneity without altering the overall material composition, the present system provides a unique platform for partially decoupling cell–matrix interactions from the bulk mechanical properties of the hydrogel. Such a design is expected to create new opportunities for regulating hMSC behavior under mechanically soft conditions that are otherwise nonpermissive for robust expansion. We demonstrate that these phase-separated Gtn-Ph/HA-Ph hydrogels, particularly the soft formulation (*G*′ = 0.5 kPa), enable long-term hMSC expansion with both enhanced proliferation and preserved multipotency over more than 15 passages. Notably, α-SMA mRNA expression remained at near-background levels throughout prolonged cultivation, indicating effective suppression of stiffness-induced fibrotic activation. This hydrogel system, therefore, provides a promising biomaterial platform for large-scale and long-term expansion of hMSCs for regenerative medicine and cell-based therapies.

## 2. Results and Discussion

### 2.1. Rational Design and Fabrication of Gtn-Ph/HA-Ph Phase-Separated Hydrogels

**Figure 1:**
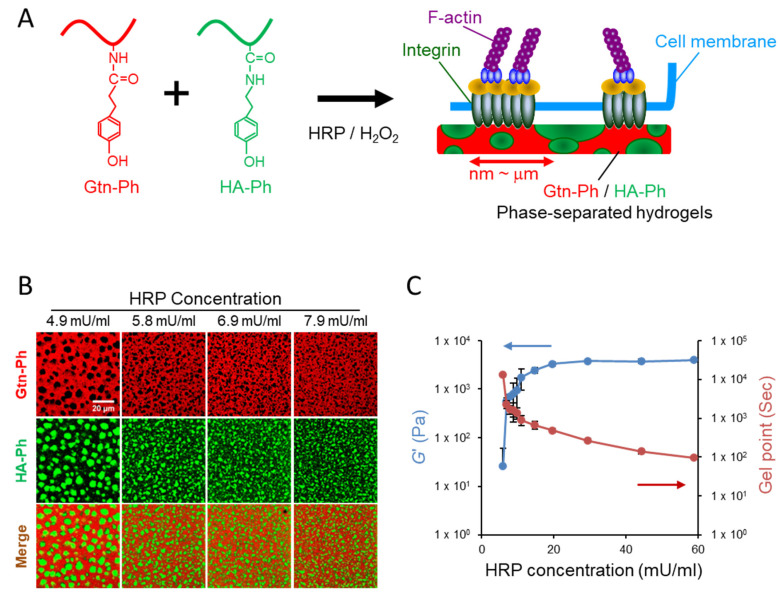
Enzyme-mediated fabrication of phase-separated Gtn-Ph/HA-Ph composite hydrogels. (**A**) A schematic illustration of the phase-separated hydrogel designed to provide decoupled biochemical and mechanical cues for hMSC culture. The hydrogel consists of micrometer-scale HA-rich domains embedded within a continuous Gtn-rich network. (**B**) Confocal laser scanning microscopy images of fluorescence-labeled hydrogels. Red fluorescence corresponds to rhodamine B–labeled Gtn-Ph (Rho-Gtn-Ph), while green fluorescence indicates fluorescein-labeled HA-Ph (FITC-HA-Ph), revealing clear phase-separated microdomains. Scale bar = 20 µm. (**C**) Storage modulus (*G*′) and gel point of hydrogels prepared with varying HRP concentrations, demonstrating kinetic control over mechanical stiffness and microstructural development. Arrows indicate the corresponding y-axes (blue: left; red: right). Data are presented as the mean ± standard deviation (SD) (*n* = 3–6).

We previously synthesized gelatin–phenol (Gtn-Ph) and hyaluronic acid–phenol (HA-Ph) conjugates via carbodiimide-mediated coupling of hydroxyphenylpropionic acid and tyramine, respectively, and demonstrated that their gelation kinetics and mechanical properties can be independently regulated by adjusting the concentrations of horseradish peroxidase (HRP) and hydrogen peroxide (H_2_O_2_) [54,55,56,57,58]. This enzyme-mediated crosslinking system enables the formation of cytocompatible hydrogels under mild conditions. However, when used as homogeneous soft matrices, Gtn-Ph hydrogels provide insufficient mechanical cues to support robust hMSC proliferation, despite being favorable for maintaining multipotency under low-stiffness conditions [54]. To overcome this limitation, we sought to introduce micrometer-scale spatial heterogeneity into soft hydrogels by embedding discrete, HA-rich domains within a continuous, integrin-adhesive Gtn-rich network. In the native stem cell niche, HA serves as a key extracellular matrix component that regulates stemness and self-renewal through interactions with CD44 receptors on hMSCs [42,43,44]. We therefore incorporated HA-Ph into the Gtn-Ph network to recapitulate both biochemical and mechanical features of the native microenvironment while maintaining a globally soft stiffness (Figure 1A). We hypothesized that the continuous Gtn-rich network would provide localized integrin-mediated adhesion sites to promote cell spreading and proliferation, whereas dispersed HA-rich domains would locally attenuate cytoskeletal tension, thereby preserving stemness.

Phase-separated Gtn-Ph/HA-Ph hydrogels were fabricated by enzyme-mediated crosslinking of mixed Gtn-Ph and HA-Ph conjugates at a 90:10 volume ratio. To visualize phase separation, fluorescein-labeled HA-Ph (Fl-HA-Ph) and rhodamine B–labeled Gtn-Ph (Rho-Gtn-Ph) were employed. The H_2_O_2_ concentration was fixed at 2.2 mM, while the HRP concentration was systematically varied to modulate gelation kinetics, with higher HRP concentrations accelerating the enzymatic crosslinking reaction. Confocal laser scanning microscopy revealed well-defined, micrometer-scale phase-separated domains, consisting of discrete Fl-HA-Ph-rich regions embedded within a continuous Rho-Gtn-Ph–rich network, within the hydrogels (Figure 1B). The characteristic domain size decreased with increasing HRP concentration, indicating that faster gelation kinetically suppressed macroscopic phase separation. Consistent with this trend, the gel point, an indicator of gelation rate, was inversely correlated with HRP concentration (Figure 1C and Table 1). At low HRP concentrations, slower crosslinking allowed sufficient time for HA-rich domains to coarsen within the continuous Gtn-rich network, resulting in larger microdomains. In contrast, rapid gelation at higher HRP concentrations arrested phase separation at earlier stages, yielding finer microstructures. The value of *G*′ increased with HRP concentration and plateaued at concentrations above 14.7 mU/mL, reflecting saturation of crosslinking density. In comparison, single-component Gtn-Ph hydrogels exhibited homogeneous fluorescence distributions, confirming the absence of phase separation (Figure S1).

Collectively, these results demonstrate that both microstructural architecture and mechanical stiffness of Gtn-Ph/HA-Ph hydrogels can be independently tuned by regulating enzymatic crosslinking kinetics. Importantly, this micrometer-scale patterning strategy does not require photolithography or specialized microfabrication equipment, providing a simple and scalable platform for engineering biomimetic culture substrates with decoupled biochemical and mechanical cues.

### 2.2. Gtn-Ph/HA-Ph-Mediated Recovery of hMSC Proliferation on Soft Hydrogels

**Figure 2:**
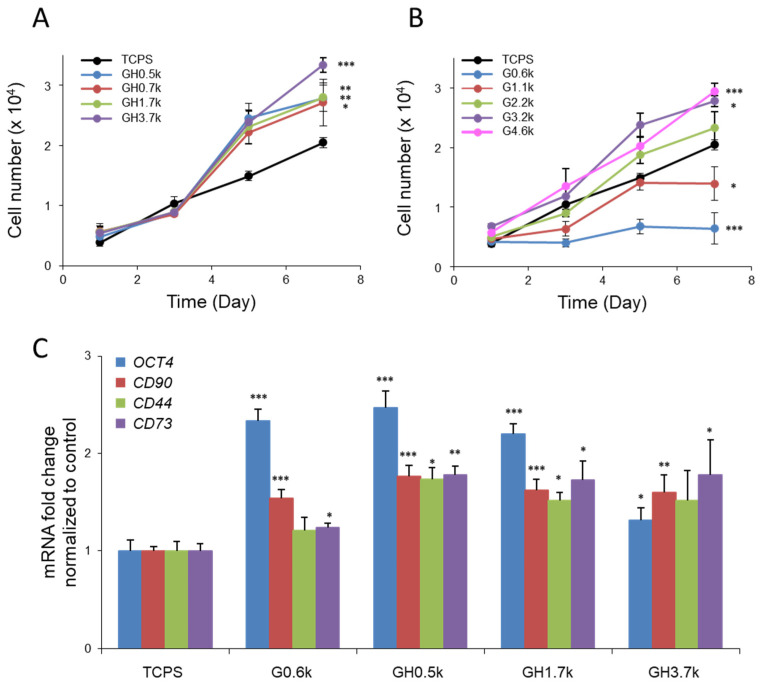
Mechanically soft yet proliferation-supportive phase-separated Gtn-Ph/HA-Ph hydrogels for hMSC culture. hMSC proliferation on (**A**) phase-separated Gtn-Ph/HA-Ph hydrogels and (**B**) homogeneous single-component Gtn-Ph hydrogels with varying stiffness was quantified from days 3 to 7 after cell seeding (*n* = 4). Statistical significance was evaluated relative to TCPS at the final time point (day 7), representing the cumulative proliferative response. (**C**) Relative mRNA expression levels of *OCT4* and hMSC surface markers *CD90*, *CD44*, and *CD73* in hMSCs cultured on Gtn-Ph/HA-Ph hydrogels at day 7. Gene expression levels were normalized to those on TCPS controls. Data are presented as mean ± standard deviation (SD) (*n* = 3). * *p* < 0.05, ** *p* < 0.01, *** *p* < 0.001.

To evaluate whether phase-separated Gtn-Ph/HA-Ph hydrogels can overcome the conventional stiffness-dependent trade-off between hMSC proliferation and stemness, we quantified cell proliferation and analyzed the expression of representative stemness-associated genes. Gtn-Ph/HA-Ph hydrogels were fabricated using HRP concentrations of 6.9, 7.8, 11, and 29.4 mU/mL, yielding *G*′ of approximately 0.5, 0.7, 1.7, and 3.7 kPa, respectively. These values closely matched those of single-component Gtn-Ph hydrogels prepared and used as controls (Table S1 and Figure S2). hMSCs were seeded onto hydrogels, and cell numbers were quantified at days 1, 3, 5, and 7. On Gtn-Ph/HA-Ph hydrogels, hMSC proliferation was significantly enhanced at days 5 and 7 compared with conventional TCPS, with cell numbers approximately 1.2–1.5-fold higher by day 7 (Figure 2A). Notably, proliferation on Gtn-Ph/HA-Ph hydrogels was largely independent of bulk substrate stiffness. Even the softest formulation (0.5 kPa; GH0.5k) supported robust cell growth. This behavior is in sharp contrast to homogeneous single-component Gtn-Ph hydrogels of comparable stiffness (~0.6 kPa), which almost completely failed to support hMSC proliferation (Figure 2B). Consistent with established mechanobiological principles, proliferation on homogeneous Gtn-Ph hydrogels increased monotonically with stiffness and plateaued above approximately 3.2 kPa, whereas very soft substrates strongly suppressed cell growth. These results are in agreement with previous reports attributing limited proliferation on soft substrates to insufficient integrin engagement, reduced actomyosin contractility, and attenuated mechanotransduction signaling [29,30,31].

The ability of GH0.5k to support robust hMSC proliferation despite its low bulk stiffness, therefore, cannot be explained by bulk mechanics alone. A plausible explanation is that the micrometer-scale, continuous Gtn-rich network within the phase-separated hydrogels may provide localized adhesive regions that are permissive for integrin engagement. Although both the Gtn-Ph and HA-Ph phases are enzymatically crosslinked and exhibit comparable bulk stiffness, the continuous Gtn-rich network is expected to present a higher local density of integrin-binding motifs within a well-connected polymer framework. Such structural features could support cell adhesion and spreading and thereby facilitate localized force transmission at the cell–matrix interface. In contrast, the HA-rich domains, which provide fewer adhesion sites, may locally attenuate cytoskeletal tension. Further studies incorporating direct visualization of cell–matrix interactions will be important for clarifying how focal adhesion organization and cytoskeletal architecture are modulated by the phase-separated microenvironment. To examine the influence of substrate properties on stem cell characteristics, we quantified the mRNA expression of *OCT4*, *CD44*, *CD73*, and *CD90*. On homogeneous Gtn-Ph hydrogels, *OCT4* expression was significantly elevated on softer hydrogels and gradually decreased with increasing stiffness (Figure S3), consistent with previous reports that soft matrices favor stem-like phenotypes [25,59]. *CD90* followed a similar trend, whereas *CD44* and *CD73* expression levels were relatively insensitive to stiffness. In contrast, on GH0.5k, *OCT4* and *CD90* expression levels were comparable to those observed on soft Gtn-Ph hydrogels, indicating preservation of stemness despite the recovery of proliferation (Figure 2C). Notably, *CD44* and *CD73* expression levels were significantly higher on all Gtn-Ph/HA-Ph hydrogels than on their Gtn-Ph-only counterparts, regardless of stiffness. Given that HA is a known ligand for CD44, this enhancement likely reflects increased HA–CD44 interactions within HA-rich microdomains, which have been implicated in the maintenance of stemness and protection against senescence-associated phenotypic drift.

Taken together, these results demonstrate that phase-separated Gtn-Ph/HA-Ph hydrogels uniquely enable the coexistence of robust hMSC proliferation and high stemness-associated gene expression under soft mechanical conditions. The ability of GH0.5k to circumvent the intrinsic limitations of conventional homogeneous soft substrates highlights the importance of microstructural design in decoupling cell proliferation from bulk substrate stiffness, thereby providing a practical solution to the long-standing proliferation–multipotency dilemma in hMSC culture.

### 2.3. Long-Term Expansion and Maintenance of Multipotency of GH0.5k Phase-Separated Hydrogels

**Figure 3:**
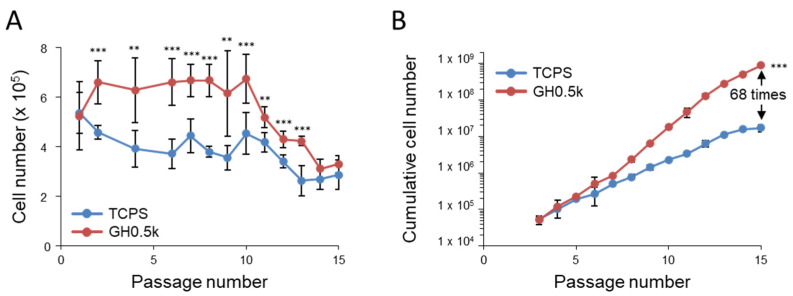
Mechanically soft phase-separated hydrogels (GH0.5k) markedly enhance long-term hMSC expansion. (**A**) hMSC numbers recovered at each passage during long-term culture on GH0.5k phase-separated hydrogels or TCPS under 10% FBS-containing conditions. (**B**) Cumulative cell yield calculated from passage-by-passage cell numbers on each substrate. Data are presented as mean ± standard deviation (SD) (*n* = 3–6). Statistical significance was evaluated relative to TCPS (** *p* < 0.01, *** *p* < 0.001).

To assess the long-term biological performance of hMSCs cultured on GH0.5k, we evaluated passage-dependent cell proliferation, cumulative cell yield, stemness-associated gene expression, and differentiation capacity over extended culture periods. hMSCs (2.4 × 10^5^ cells) were seeded onto GH0.5k or conventional TCPS in 10 cm dishes and passaged every 3 days. Experiments were conducted under two culture conditions: a medium containing 10% fetal bovine serum (FBS) and an animal component-free (ACF) medium. Under 10% FBS conditions, hMSC proliferation on GH0.5k was significantly higher than on TCPS up to passage 13 (Figure 3A). Between passages 2 and 10, 1.2–1.5-fold more cells were recovered from GH0.5k than from TCPS, consistent with the short-term proliferation results. Although proliferation gradually declined on both substrates after passage 10, long-term expansion on GH0.5k was sustained up to passage 15, whereas TCPS-supported proliferation declined markedly at earlier passages. As a result, the cumulative cell yield after 15 passages on GH0.5k was approximately 68-fold greater than that on TCPS (Figure 3B).

**Figure 4:**
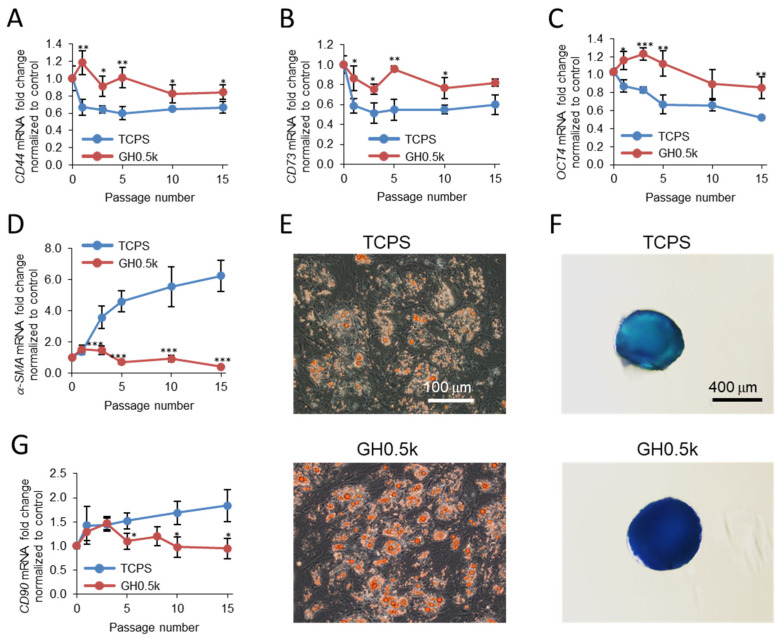
Soft GH0.5k phase-separated hydrogels support long-term maintenance of hMSC multipotency under 10% FBS-containing conditions. (**A**–**D**) Relative mRNA expression levels of *CD44*, *CD73*, *CD90*, *OCT4*, and α-SMA in hMSCs cultured on GH0.5k hydrogels, compared with those cultured on TCPS, up to passage 15. Data are presented as mean ± standard deviation (SD) (*n* = 3). * *p* < 0.05, ** *p* < 0.01, *** *p* < 0.001. (**E**,**F**) Representative lineage differentiation of hMSCs expanded on GH0.5k hydrogels, followed by transfer to 24-well plastic plates for induction. Cells were differentiated toward (**E**) adipogenic and (**F**) chondrogenic lineages using the corresponding induction media. Adipogenesis and chondrogenesis were evaluated by Oil Red O staining of intracellular lipid droplets and Alcian Blue staining of cartilage matrix aggrecan, respectively. Scale bars: (**E**) 100 μm, (**F**) 400 μm. (**G**) Relative mRNA expression levels of *CD90* in hMSCs cultured on GH0.5k hydrogels, compared with those cultured on TCPS. Data are presented as mean ± standard deviation (SD) (*n* = 3). * *p* < 0.05.

Gene expression analysis revealed that hMSCs expanded on GH0.5k maintained significantly higher levels of *CD44*, *CD73*, and *OCT4* compared with TCPS controls throughout long-term culture (Figure 4A–C). Importantly, α-SMA mRNA expression remained at near-background levels on GH0.5k throughout all 15 passages, whereas it was markedly upregulated on TCPS (Figure 4D). Given that α-SMA is a well-established marker of myofibroblastic activation, cellular senescence, and loss of regenerative potential in hMSCs [22,23,24], these results indicate that GH0.5k effectively suppresses stiffness-induced fibrotic and senescent phenotypes during prolonged expansion. Consistent with the preserved stemness profile, lineage differentiation assays revealed that hMSCs expanded on GH0.5k retained robust adipogenic and chondrogenic differentiation capacities after 15 passages (Figure 4E,F), as evidenced by enhanced intracellular lipid accumulation and extracellular aggrecan deposition relative to TCPS-expanded cells. In contrast, alkaline phosphatase staining indicated comparable osteogenic differentiation between GH0.5k- and TCPS-expanded cells (Figure S4). This observation is likely attributable to mechanical memory effects, whereby prior exposure to stiff substrates predisposes hMSCs toward osteogenic commitment even after transfer to differentiation conditions [23,60]. Supporting this interpretation, the expression level of *CD90*, one of the key regulators of osteogenic differentiation [61], was slightly higher in TCPS-expanded cells than in those cultured on GH0.5k (Figure 4G). Previous studies have shown that mechanical memory acquired on stiff culture substrates can be progressively relaxed during subsequent culture on soft matrices [60]. Although the underlying mechanisms remain to be elucidated, the persistent suppression of α-SMA expression observed on GH0.5k is consistent with such relaxation of stiffness-induced cellular activation. Together, these findings demonstrate that GH0.5k phase-separated hydrogels enable sustained long-term expansion of hMSCs while preserving multipotency and suppressing stiffness-induced dysfunction. By providing a mechanically protective yet proliferation-supportive microenvironment, GH0.5k maintains functional differentiation capacity over extended culture, highlighting its potential as a scalable platform for hMSC manufacturing. It should be noted that, in the long-term expansion experiments, hMSCs were routinely passaged every 3–7 days and therefore were not continuously maintained on a single hydrogel surface. Accordingly, the proliferation data reflect repeated short-term culture on freshly prepared Gtn-Ph/HA-Ph hydrogels rather than uninterrupted long-term residence on one substrate. In addition, the storage stability of the Gtn-Ph/HA-Ph hydrogels was evaluated, and the hydrogels retained structural integrity for at least 180 days after gelation (Figure S5).

**Figure 5:**
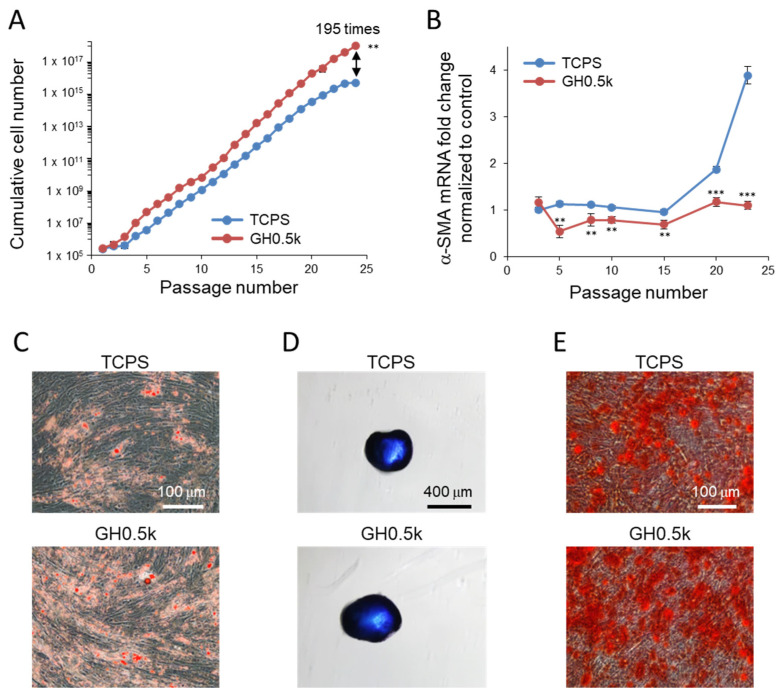
Long-term culture of hMSCs on a mechanically soft, phase-separated GH0.5k hydrogel under ACF conditions. (**A**) Cumulative hMSC numbers on GH0.5k and conventional TCPS were quantified up to passage 24. All data are presented as mean ± SD (*n* = 3–6) (** *p* < 0.01). (**B**) Relative mRNA expression of α-SMA in hMSCs cultured on GH0.5k, normalized to control TCPS. All data are presented as mean ± SD (*n* = 3) (** *p* < 0.01, *** *p* < 0.001). (**C**–**E**) Representative lineage differentiation of hMSCs maintained in ACF medium. (**C**) Adipogenic, (**D**) chondrogenic, and (**E**) osteogenic differentiation were assessed by staining intracellular lipid droplets with Oil Red O, cartilage-specific aggrecan with Alcian Blue, and deposited calcium with Alizarin Red S, respectively. Scale bars: (**C**) 100 μm, (**D**) 400 μm, (**E**) 100 μm.

Long-term performance was further evaluated under ACF conditions. Under ACF culture, hMSC proliferation was markedly enhanced on both substrates, consistent with previous reports demonstrating higher proliferation rates in chemically defined serum-free media [18,19,20,21]. Nevertheless, the cumulative cell yield on GH0.5k reached approximately 195-fold higher than that on TCPS after 24 passages (Figure 5A). Stemness marker expression was broadly similar between the two substrates under ACF conditions (Figure S6), indicating that the ACF medium itself promotes maintenance of stemness-related transcriptional profiles.

Although the differences in several stemness-related markers between substrates became less pronounced under ACF conditions, α-SMA expression remained strongly substrate-dependent under both culture conditions. In contrast, α-SMA mRNA expression increased markedly on TCPS, particularly after passage 15, whereas it remained at near-background levels on GH0.5k throughout all 24 passages under both FBS-containing and ACF conditions (Figure 4D and Figure 5B). These observations suggest that biochemical signals provided by the culture medium can partially influence stemness-related markers, whereas cytoskeletal activation reflected by α-SMA expression is primarily governed by the mechanical microenvironment provided by the soft, phase-separated GH0.5k hydrogel.

Recent studies have identified α-SMA upregulation not only as a marker of myofibroblastic transition but also as a hallmark of cellular senescence in hMSCs, where age- or passage-related cytoskeletal remodeling drives the acquisition of a contractile, fibroblast-like phenotype [23,62]. The complete suppression of α-SMA mRNA expression on GH0.5k therefore indicates that this soft, phase-separated hydrogel effectively protects hMSCs from stiffness-induced senescence during long-term expansion. Differentiation assays performed under ACF conditions further confirmed that hMSCs expanded on GH0.5k retained robust multipotency (Figure 5C–E and Figure S7). Adipogenic lipid accumulation, chondrogenic aggrecan deposition, and osteogenic calcium mineralization were all clearly preserved, with particularly strong adipogenic differentiation observed. Although ACF medium supported the maintenance of stemness-associated gene expression and osteogenic and chondrogenic differentiation potential on both substrates, the stiff TCPS substrate failed to suppress α-SMA mRNA upregulation or maintain adipogenic differentiation capacity. Notably, only GH0.5k enabled the simultaneous preservation of stemness, multilineage differentiation capacity, and suppression of α-SMA mRNA expression under ACF conditions.

Collectively, these results demonstrate that GH0.5k phase-separated Gtn-Ph/HA-Ph hydrogels support sustained hMSC proliferation while preserving multipotency during long-term expansion under both serum-containing and animal component-free conditions. By combining microscale adhesive patterning with a globally soft mechanical environment, this hydrogel system effectively suppresses senescence-associated cytoskeletal activation and maintains multilineage differentiation capacity, highlighting its potential as a scalable platform for long-term hMSC manufacturing.

## 3. Materials and Methods

### 3.1. Materials

Gelatin (Gtn; *M*_w_ = 80–140 kDa) and horseradish peroxidase (HRP) were purchased from Wako Pure Chemical Industries (Osaka, Japan). Sodium hyaluronic acid (HA; *M*_w_ = 90 kDa) was kindly provided by JNC Corporation (Tokyo, Japan). Hydrogen peroxide (H_2_O_2_) was obtained from Lancaster (Ward Hill, MA, USA). Hydroxyphenylpropionic acid (HPA), tyramine hydrochloride (Tyr·HCl), N-hydroxysuccinimide (NHS), 1-ethyl-3-(3-dimethylaminopropyl) carbodiimide hydrochloride (EDC·HCl), rhodamine B amine, 5-aminofluorescein, polyethylene glycol (PEG; Mw = 2 kDa), 5-bromo-4-chloro-3-indolyl phosphate/nitro blue tetrazolium (BCIP/NBT) tablets, alizarin red S, alcian blue 8GX, and oil red O were purchased from Sigma-Aldrich (St. Louis, MO, USA). Trypsin/EDTA (0.25%/1 mM) and phosphate-buffered saline (PBS) were obtained from Thermo Fisher Scientific (Waltham, MA, USA). Acetic acid and 2-propanol were purchased from VWR International (Radnor, PA, USA) and Merck & Co. (Rahway, NJ, USA), respectively. MSCGM™ medium containing 10% fetal bovine serum (FBS) was obtained from Lonza (Basel, Switzerland). Animal component-free (ACF) medium (MesenCult™-ACF) and ACF cell dissociation kits were purchased from STEMCELL Technologies Inc. (Vancouver, BC, Canada). Bone marrow-derived human mesenchymal stem cells (hMSCs) from Lonza (PT-2501, lot no. 0000423370). Adipogenic, osteogenic, and chondrogenic induction media were purchased from PromoCell GmbH (Heidelberg, Germany). Trypan blue solution (0.4%), the Quant-iT™ PicoGreen^®^ dsDNA assay kit, TRIzol™ reagent, and TaqMan^®^ PCR primers were purchased from Life Technologies (Carlsbad, CA, USA).

### 3.2. Synthesis of Gtn-Ph and HA-Ph, and Their Fluorescence Labeling

Gtn-Ph and HA-Ph conjugates were synthesized via carbodiimide-mediated coupling reactions following previously established procedures with minor modifications [54,55,56,57]. Rhodamine-labeled Gtn-Ph (Rho-Gtn-Ph) and fluorescein-labeled HA-Ph (Fl-HA-Ph) were prepared by coupling rhodamine B amine or 5-aminofluorescein to the corresponding phenol-modified polymers according to a previous report [56]. The resulting conjugates were purified by extensive dialysis against deionized water and subsequently lyophilized. Detailed synthetic procedures and characterization are provided in the Supplementary Materials.

### 3.3. Rheological Measurement of Hydrogels

The rheological properties of the hydrogels during gelation were evaluated using a HAAKE Rheoscope 1 rheometer (Karlsruhe, Germany) equipped with a cone-and-plate geometry (35 mm diameter, 0.949° cone angle) [56]. Measurements were conducted at 37 °C under oscillatory shear with a constant strain of 1% and a frequency of 1 Hz. A roughened glass plate was employed as the lower surface to prevent sample slippage. A 5.0 wt% solution of Gtn-Ph or Gtn-Ph/HA-Ph (90:10, *v*/*v*) in PBS was mixed sequentially with predetermined concentrations of HRP and H_2_O_2_. The mixture was briefly vortexed and immediately loaded onto the rheometer plate. The upper cone was lowered to a measurement gap of 0.024 mm, and a thin layer of silicone oil was applied around the periphery to minimize solvent evaporation during measurement. The storage modulus (*G*′) and loss modulus (*G*″) were recorded as functions of time. The gelation point was defined as the time at which *G*′ and *G*″ intersected. The apparent Young’s modulus (*E*′) was estimated from *G*′ using the following relation:

$$E^{\prime }\;=\;2G^{\prime }\;\left(1\;+\;\nu \right)$$

assuming an incompressible isotropic hydrogel with a Poisson’s ratio (*ν*) of 0.5 [63,64]. All measurements were performed in triplicate.

### 3.4. Observation of Micro-Pattern in Phase-Separated Hydrogels

To visualize phase separation between Gtn-Ph and HA-Ph within phase-separated hydrogels, rhodamine-labeled Gtn-Ph (Rho-Gtn-Ph) and fluorescein-labeled HA-Ph (Fl-HA-Ph) were used as fluorescent probes. Each conjugate was dissolved separately in PBS at 5.0 wt% and thoroughly mixed. A 90:10 (*v*/*v*) mixture was prepared by combining 225 µL of Rho-Gtn-Ph solution with 25 µL of Fl-HA-Ph solution. Appropriate volumes of HRP and H_2_O_2_ solutions at predetermined concentrations were sequentially added to 250 µL of the Rho-Gtn-Ph/Fl-HA-Ph mixture. The solution was briefly vortexed and immediately dispensed into glass-bottom dishes (MatTek Corp., Ashland, MA, USA). Gelation was carried out by incubation at 37 °C overnight in a humidified 5% CO_2_ incubator. The resulting hydrogels were observed using a Zeiss LSM 5 DUO confocal laser scanning microscope (Carl Zeiss, Jena, Germany). Fluorescence images were acquired to visualize the spatial distribution of Gtn-rich (red channel) and HA-rich (green channel) microdomains.

### 3.5. Long-Term hMSC Culture

Long-term expansion assays were performed using bone marrow-derived hMSCs. Gtn-Ph/HA-Ph hydrogels were prepared in 10 cm culture dishes. hMSCs (2.4 × 10^5^ cells per dish) were seeded and cultured in either MSCGM™ containing 10% FBS or MesenCult™-ACF medium. Cells were passaged every 3 days using 0.25% trypsin-EDTA for FBS-containing cultures and the ACF Cell Dissociation Kit for ACF cultures. At each passage, harvested cells were counted using a hemocytometer and reseeded at a density of 2.4 × 10^5^ cells per 10 mL onto freshly prepared GH0.5k hydrogels. The cumulative cell number at passage *n* was calculated according to the following equation [14]:

$$N_{n}=\;\left(\frac{N_{c}}{2.4\;\times \;10^{5}}\right)\times \;N_{n-1}$$

where *Nₙ* is the cumulative cell number at passage *n*, and *N_c_* is the number of cells harvested at that passage. The initial value *N*_0_ was defined as 2.4 × 10^5^ cells.

### 3.6. Statistical Analysis

All quantitative data are presented as the mean ± standard deviation (SD). Statistical analyses were performed using a two-tailed Student’s *t*-test. A *p*-value < 0.05 was considered statistically significant.

## 4. Conclusions

In this study, we developed phase-separated Gtn-Ph/HA-Ph hydrogels as a novel biomaterial platform to overcome the long-standing challenge of balancing proliferation and multipotency during large-scale expansion of hMSCs. On single-component homogeneous Gtn-Ph hydrogels, hMSCs exhibited a classical stiffness-dependent trade-off; soft substrates (~0.6 kPa) preserved stemness but limited proliferation, whereas stiffer substrates enhanced proliferation at the expense of multipotency. By introducing micrometer-scale phase separation between dot-like HA-rich domains and a continuous Gtn-rich network, we successfully created a biomimetic microenvironment that decouples local adhesive signaling from global substrate stiffness. As a result, the optimized soft formulation, GH0.5k, supported robust hMSC proliferation while maintaining high expression levels of stemness-associated markers. Importantly, the senescence- and fibrosis-associated marker α-SMA was suppressed to near-background levels throughout long-term culture under both serum-containing and ACF conditions. hMSCs expanded on GH0.5k retained strong adipogenic, chondrogenic, and osteogenic differentiation potentials even after extended passaging, confirming the preservation of multipotency. Under ACF conditions, the cumulative cell yield on GH0.5k reached approximately 195-fold higher than that on conventional TCPS after 24 passages, demonstrating the outstanding scalability of this system. Collectively, these results demonstrate that phase-separated Gtn-Ph/HA-Ph hydrogels constitute a powerful and versatile culture platform that simultaneously supports long-term hMSC proliferation while maintaining multipotency. This hydrogel design offers a promising strategy for the development of clinically relevant and scalable hMSC manufacturing processes for regenerative medicine and cell-based therapies.

## Figures and Tables

**Table 1:** Rheological properties of Gtn-Ph/HA-Ph phase-separated hydrogels ^a^.

Sample	Gtn-Ph (wt%)	HA-Ph (wt%)	HRP (mU/mL)	H_2_O_2_ (mM)	*G*′ (kPa)	*E*′ (kPa) ^b^	Gel Point (min) ^c^
GH0.5k	4.5	0.5	6.9	2.2	0.54 ± 0.10	1.63 ± 0.30	337 ± 8.0
GH0.7k	4.5	0.5	7.8	2.2	0.70 ± 0.08	2.09 ± 0.23	27.7 ± 6.8
GH0.8k	4.5	0.5	8.8	2.2	0.79 ± 0.52	2.36 ± 1.57	27.0 ± 13.8
GH1.0k	4.5	0.5	9.8	2.2	0.96 ± 0.72	2.87 ± 2.15	22.95 ± 7.2
GH1.7k	4.5	0.5	11.0	2.2	1.74 ± 0.80	5.21 ± 2.40	15.0 ± 4.2
GH2.4k	4.5	0.5	14.7	2.2	2.44 ± 0.35	7.33 ± 1.05	11.2 ± 2.5
GH3.3k	4.5	0.5	19.6	2.2	3.27 ± 0.35	9.81 ± 1.04	8.06 ± 0.9
GH3.7k	4.5	0.5	29.4	2.2	3.73 ± 0.27	11.2 ± 0.81	4.45 ± 0.55
GH3.9k	4.5	0.5	58.8	2.2	3.92 ± 0.32	11.8 ± 0.96	1.61 ± 0.13
GH3.8k	4.5	0.5	88.0	2.2	3.80 ± 0.37	11.4 ± 1.11	1.21 ± 0.22

^a^ Measurement was taken with constant deformation of 1% at 1 Hz and 37 °C (*n* = 3–5). Results are shown as the average values and standard deviation. ^b^ The value of Young’s modulus (*E*′) was estimated from the storage modulus (*G*′). ^c^ Gel point is defined as the time at which the crossover of *G*′ and loss modulus (*G*″) occurs. Herein, it is used as an indicator of the rate of gelation.

## Data Availability

The original contributions presented in this study are included in the article/Supplementary Material. Further inquiries can be directed to the corresponding author.

## Supplementary Materials

The following supporting information can be downloaded at: https://www.mdpi.com/article/10.3390/ijms27072932/s1.
