# Supplementary material for: Mechanically Soft Phase-Separated Gelatin/Hyaluronic Acid Hydrogels Support Long-Term Expansion of Human Mesenchymal Stem Cells While Preserving Multipotency
Source: Int J Mol Sci. 2026 Mar 24;27(7):2932. doi: 10.3390/ijms27072932 (PMC13073967; doi:10.3390/ijms27072932)
Supplement: Supplementary file 1 [file ijms-27-02932-s001.zip › ijms-4154241-supplementary.pdf]

Supplementary Materials

for

Mechanically soft phase-separated gelatin/hyaluronic acid hydrogels support long-term expansion of human mesenchymal stem cells while preserving multipotency

Atsushi Yamashita[1,2], Nunnarpas Yongvongsoontorn[2], Joo Eun Chung[2] and Motoichi Kurisawa[1,2]*

[1]Institute of Bioengineering and Bioimaging, 31 Biopolis Way, The Nanos, 138669, Singapore

[2]Graduate School of Advanced Science and Technology, Japan Advanced Institute of Science and Technology, 1-1 Asahidai, Nomi, Ishikawa 923-1292, Japan

*Correspondence: kurisawa@jaist.ac.jp

Supplementary Methods

S1. Synthesis of Gtn-Ph and HA-Ph conjugates

Gelatin-phenol (Gtn-Ph) conjugates were synthesized via carbodiimide/NHS-mediated coupling reaction, following previously reported protocols [52,53]. Briefly, hydroxyphenylpropionic acid (HPA) (33.2 g, 200 mmol) was dissolved in a 3:2 (v/v) mixture of distilled water and N,N-dimethylformamide (DMF) (2.5 L) under stirring. N-hydroxysuccinimide (NHS) (32 g, 280 mmol) and 1-ethyl-3-(3-dimethylaminopropyl) carbodiimide hydrochloride (EDC·HCl) (38.2 g, 200 mmol) were added to activate the HPA carboxyl groups, and the pH was maintained at 4.7 throughout the activation reaction at room temperature for 5 h. An aqueous gelatin solution (1.5 L, 6.67 wt%) was then added slowly, and the coupling reaction was allowed to proceed overnight at room temperature. The reaction mixture was subsequently adjusted to pH 7.0, transferred into a

dialysis tube (MWCO 3,500 Da), and dialyzed sequentially against 100 mM NaCl (3 days), distilled water/ethanol (3:1, v/v; 2 days), and distilled water (3 days). The purified solution was lyophilized to yield Gtn-Ph as a white porous solid.

Hyaluronic acid-phenol (HA-Ph) conjugates were similarly synthesized via a carbodiimide/NHS-mediated coupling reaction [54]. HA (20 g) was dissolved in 2.0 L of distilled water under stirring. Tyr·HCl (4.05 g, 23 mmol), followed by NHS (6.4 g, 56 mmol) and EDC·HCl (9.6 g, 50 mmol), were then added to initiate carbodiimide-mediated coupling. The reaction proceeded overnight at room temperature while maintaining the pH at 4.7 using 6 M NaOH. The mixture was then adjusted to pH 7.0, transferred into a dialysis tube (MWCO 3,500 Da), and dialyzed against the same sequence of purification solutions as described above. The resulting HA-Ph conjugate was obtained as a white, porous solid after lyophilization and stored at −20 °C.

S2. Synthesis of fluorescence-labeled Gtn-Ph and HA-Ph conjugates

For rhodamine-labelled Gtn-Ph (Rho-Gtn-Ph), Gtn-Ph (1 g, 2.5 mmol) was dissolved in distilled water (40 mL) and purged with nitrogen for 1 h. Rhodamine B amine (81 mg, 0.23 mmol) dissolved in dimethyl sulfoxide (DMSO) (1.62 mL) was added, followed by NHS (290 mg, 2.5 mmol) and EDC·HCl (479 mg, 2.5 mmol). The pH was maintained at 4.7 during the reaction, which proceeded overnight at room temperature. After adjusting the pH to 7.0, the reaction mixture was filtered to remove unreacted dye and dialyzed using the same purification sequence as above (MWCO 3,500 Da). The resulting Rho-Gtn-Ph was obtained as a light pink, porous solid after lyophilization. The degree of dye conjugation was quantified spectrophotometrically at 490 nm using rhodamine B calibration standards.

For fluorescein-labeled HA-Ph (Fl-HA-Ph), HA (1 g, 2.5 mmol) was dissolved in distilled water (100 mL) and purged with nitrogen for 1 h. Tyramine hydrochloride (162 mg, 0.93 mmol) and 5-aminofluorescein (81 mg, 0.23 mmol in 1.62 mL DMSO) were added, followed by NHS (290 mg, 2.5 mmol) and EDC·HCl (479 mg, 2.5 mmol). The pH was adjusted to 4.7 during the overnight reaction, then to pH 7.0 afterward. Filtration and dialysis were performed as described for Rho-Gtn-Ph, and the final Fl-HA-Ph conjugate was obtained by lyophilization. Dye conjugation efficiency was quantified by absorbance measurements at 490 nm using 5-aminofluorescein standards [54].

S3. Stability of Gtn-Ph/HA-Ph Hydrogels

To assess the stability of Gtn-Ph/HA-Ph, hydrogels were prepared as described above and stored at either 4 °C or room temperature for up to 180 days after gelation. At predetermined time points, hydrogels were extensively washed with PBS to remove unreacted or loosely associated components. The washed hydrogels were then lyophilized and weighed. No detectable polymer loss was observed after repeated washing at any time point, indicating that the hydrogels retained structural integrity during storage.

S4. Quantification of human mesenchymal stem cell (hMSC) proliferation on conventional tissue culture polystyrene (TCPS) and hydrogels

To evaluate the proliferation of hMSCs (Lonza, PT-2501, lot no. 0000423370; donor age, 23 years) on different substrates, Gtn-Ph and Gtn-Ph/HA-Ph composite hydrogels were prepared in 24-well plates. The hydrogels were pre-equilibrated by soaking in 0.75 mL of FBS-containing medium (MSCGM™) overnight at 37 °C. Before cell seeding, 0.75 mL of the medium was

aspirated, and hMSCs (5×10^3 cells/well) suspended in 0.5 mL of fresh MSCGM™ medium were gently added onto the hydrogel surfaces. Cell cultures were maintained under standard conditions (37 °C, 5% CO_2), and the medium was replaced every three days. At designated time points, cells were detached from the substrates using 0.25% trypsin/1 mM EDTA solution and collected for quantification. The total number of cells on each substrate was determined using the Quant-iT™ PicoGreen® double-stranded DNA (dsDNA) assay according to the manufacturer's instructions. Fluorescence intensity was measured with a microplate reader, and cell numbers were calculated from a standard curve generated using known hMSCs counts

S5. Analysis of mRNA expression

Total cellular RNA was extracted using TRIzol™ reagent (Invitrogen, Carlsbad, CA, USA) according to the manufacturer's instructions. The purity and concentration of RNA were confirmed spectrophotometrically by measuring the absorbance at 260 and 280 nm. Complementary DNA (cDNA) was synthesized from total RNA using a reverse transcription kit (manufacturer's protocol). Quantitative real-time polymerase chain reaction (qRT-PCR) was performed using a Bio-Rad iQ5 multicolor Real-Time PCR Detection System (Bio-Rad, Hercules, CA, USA). Each reaction (20 µL total volume) contained cDNA template, TaqMan primer probe mix, PCR Master Mix (Bioline Ltd., London, UK), and diethyl pyrocarbonate (DEPC)-treated water (Invitrogen, Carlsbad, CA, USA). Amplification was carried out under the cycling conditions recommended by the TaqMan Gene Expression Assay protocol. The relative expression level of each target gene was normalized to *glyceraldehyde-3-phosphate dehydrogenase* (*GAPDH*) as an internal reference. The results were expressed as fold changes relative to those of hMSCs cultured on polystyrene control substrates using the $2^{-\Delta\Delta Ct}$ method.

S6. Assessment of hMSCs multipotency

The multipotency of hMSCs was evaluated by assessing their adipogenic, osteogenic, and chondrogenic differentiation capacities at designated passages after expansion on the indicated substrates. For all differentiation assays, hMSCs were first harvested from the expansion substrates and subsequently re-seeded onto standard culture platforms appropriate for each lineage induction. For adipogenic differentiation, hMSCs (2.0×10^4 cells/well) were seeded onto fibronectin-coated 24-well plates. After 3 days of culture in growth medium, the medium was replaced with adipogenic induction medium. The medium was changed every 3 days for 2 weeks. Cells were then fixed using Saccomanno's fixative for 30 min and stained with Oil Red O for 30 min to visualize intracellular lipid accumulation. Following microscopic observation, Oil Red O was extracted using isopropanol, and the absorbance at 492 nm was measured to quantify lipid deposition. For osteogenic differentiation, hMSCs (2.0×10^4 cells/well) were seeded onto fibronectin-coated 24-well plates and cultured for 3–7 days. The culture medium was then replaced with osteogenic induction medium, which was changed every 3 days for 2 weeks. After fixation with Saccomanno's fixative solution, alkaline phosphatase activity or calcium deposition were evaluated by staining with 5-bromo-4-chloro-3-indolyl phosphate/nitro blue tetrazolium (BCIP/NBT) or Alizarin Red S, respectively. For quantification, Alizarin Red S was extracted with 10% cetylpyridinium chloride, and the absorbance at 584 nm was measured. For chondrogenic induction, hMSCs (1.0×10^5 cells/well) were seeded in PrimeSurface™ round-bottom 96-well plates (Sumitomo Bakelite Co. Ltd., Tokyo, Japan) to promote spheroid formation. After 3 days, the medium was replaced with chondrogenic induction medium, which was changed every 3 days for 3 weeks. Spheroids were fixed in Kahle's fixative for 10 min and stained with Alcian Blue

8GX for 45 min to detect extracellular aggrecan. The dye was then extracted using 6 M guanidine hydrochloride, and the absorbance at 595 nm was measured to quantify glycosaminoglycan production.

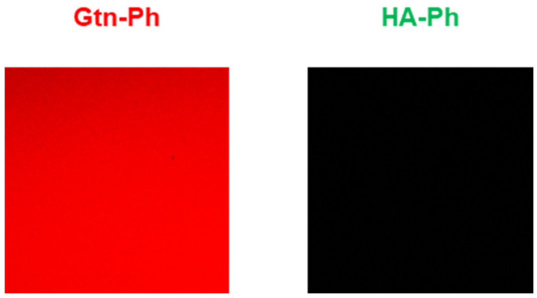

Figure S1:

Confocal laser scanning microscopic images of Rho-labeled Gtn-Ph hydrogels. The hydrogels exhibit uniform red fluorescence originating from Rho-Gtn-Ph, while the FITC channel shows no detectable signal due to the absence of FITC-HA-Ph.

Table S1 Rheological properties of Gtn-Ph hydrogels.[a]

Sample	Gtn-Ph (wt%)	HRP (mU/mL)	H_2O_2 (μM)	*G′* (kPa)	*E′* (kPa)[b]
G0.6k	5	39	1.08	0.56 ± 0.02	1.68 ± 0.05
G1.1k	5	39	1.3	1.14 ± 0.07	3.41 ± 0.22
G2.2k	5	39	1.57	2.24 ± 0.16	6.72 ± 0.49
G3.2k	5	39	1.8	3.22 ± 0.15	9.67 ± 0.45
G4.6k	5	39	2.0	4.63 ± 0.01	13.9 ± 0.01

[a]Measurement was taken with constant deformation of 1% at 1 Hz and 37 °C (n = 3-5). Results are shown as the average values and standard deviation. [b]The value of Young's modulus (*E′*) was estimated from the storage modulus (*G′*).

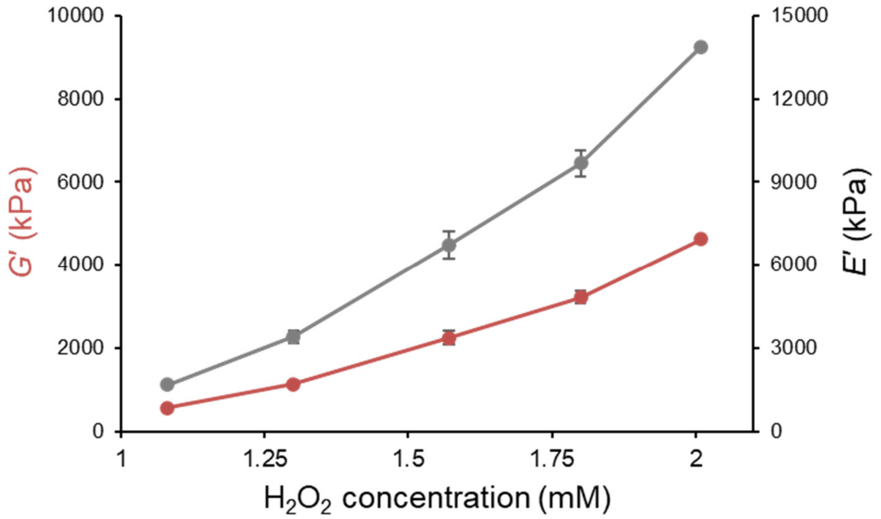

Figure S2:

Rheological characterization of Gtn-Ph hydrogels formed via enzymatic crosslinking. *G′* and estimated *E′* of hydrogels prepared with varying concentrations of H_2O_2. *E′* values were calculated from the corresponding *G′* values using the standard conversion for incompressible hydrogels. Data are presented as mean ± SD (n = 3).

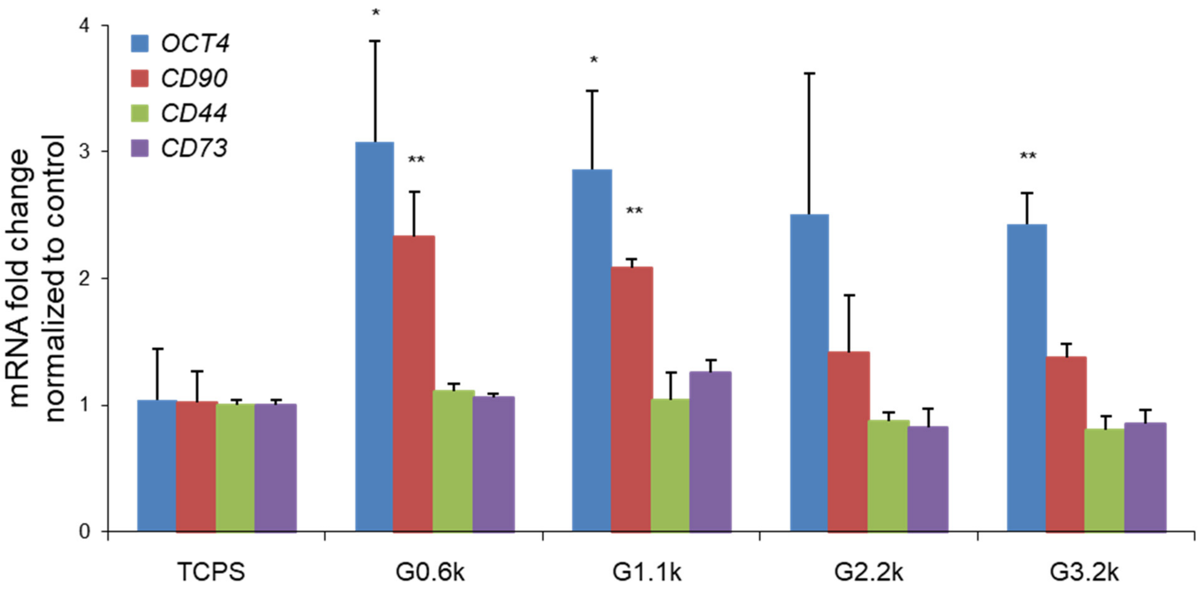

Figure S3:

Relative mRNA expression levels of *OCT4* and the hMSC markers *CD90*, *CD44*, and *CD73* on Gtn-Ph hydrogels at day 7. Expression levels were normalized to those on TCPS controls. Results are shown as mean ± SD (n = 3) (*$P < 0.05$, **$P < 0.01$).

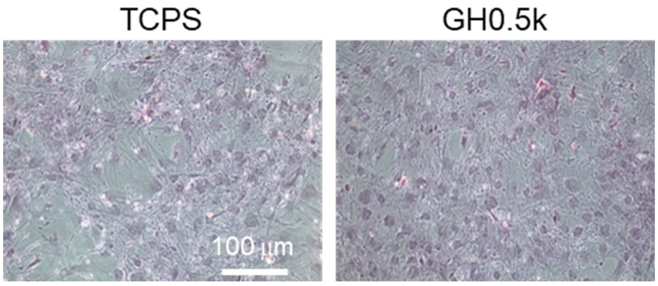

Figure S4:

Representative lineage differentiation of hMSCs expanded on GH0.5k hydrogels, followed by transfer to 24-well plastic plates for induction. Cells were differentiated toward osteogenic lineages using the corresponding induction media. Osteogenesis was evaluated by staining alkaline phosphatase with NBT/BCIP.

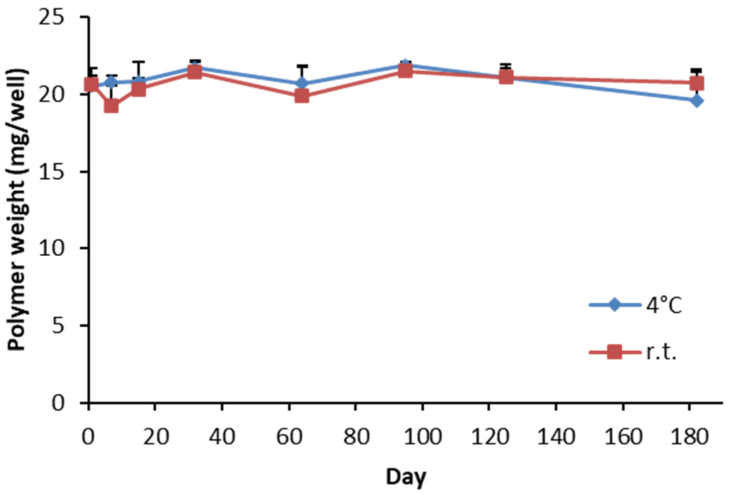

Figure S5:

Gtn-Ph/HA-Ph hydrogels were stored at 4 °C or room temperature for up to 180 days after gelation. At the indicated time points, hydrogels were extensively washed with PBS, lyophilized, and weighed to evaluate polymer retention.

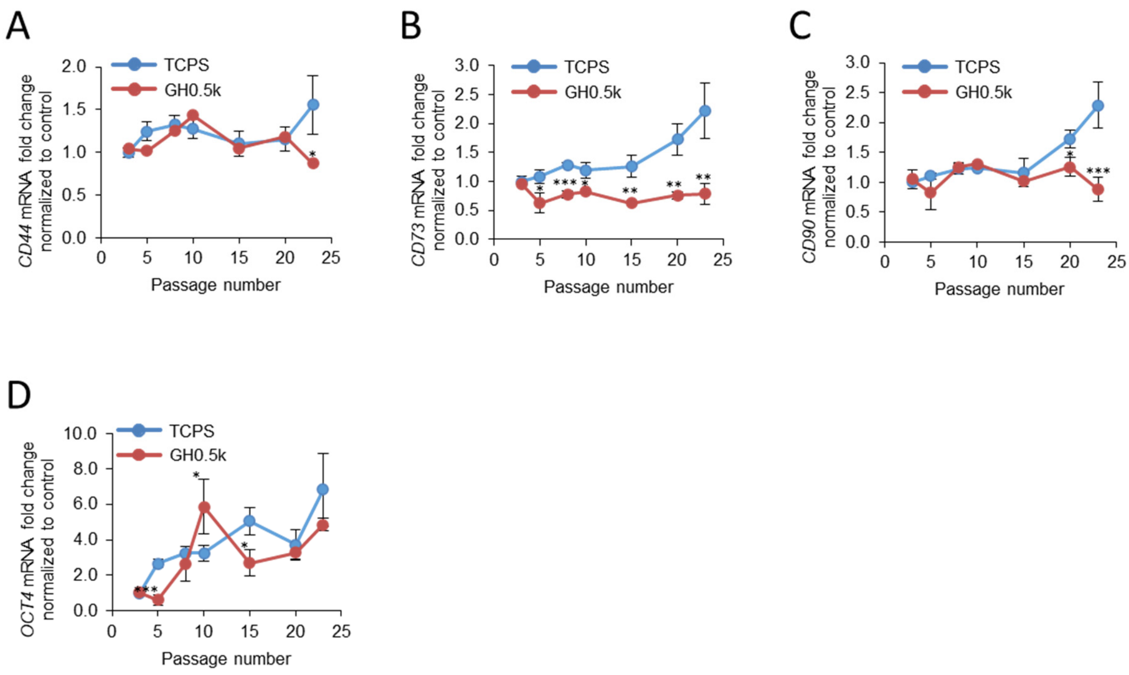

Figure S6:

Relative mRNA expression levels of stemness-associated markers in hMSCs cultured long-term on the soft GH0.5k phase-separation hydrogel under ACF conditions. The expression levels of (A) *CD44*, (B) *CD73*, (C) *CD90*, and (D) *OCT4* on GH0.5k were compared with those on control TCPS. All results are presented as mean ± SD (n = 3) (*$P < 0.05$, **$P < 0.01$, ***$P < 0.001$).

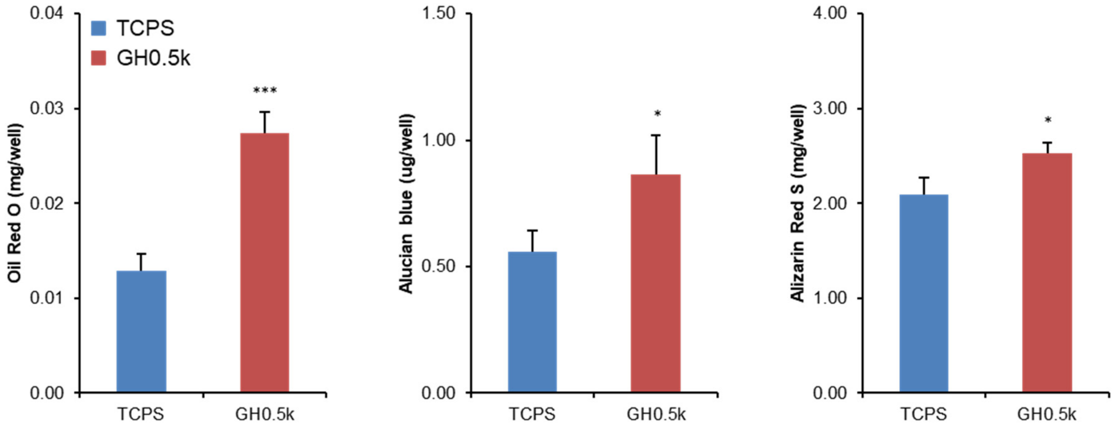

Figure S7:

Representative differentiation of hMSCs cultured with ACF medium on TCPS and GH0.5k. After transfer to new plastic plates, hMSCs were induced to undergo adipogenesis, chondrogenesis, and osteogenesis in conventional lineage induction media. Adipogenic, chondrogenic, and osteogenic differentiation were assessed by staining fat droplets with oil red O, cartilage matrix aggrecan with alcian blue, and calcium-rich nodules with alizarin red S, respectively. Amounts of oil red O, alcian blue, and alizarin red S were quantitatively analyzed by measurement of absorbance after elution of dyes. Results are shown as the average values ± standard deviation (n = 3) (*P < 0.05, ***P < 0.001).
